# Effects of cytosine methylation on transcription factor binding sites

**DOI:** 10.1186/1471-2164-15-119

**Published:** 2014-03-26

**Authors:** Yulia A Medvedeva, Abdullah M Khamis, Ivan V Kulakovskiy, Wail Ba-Alawi, Md Shariful I Bhuyan, Hideya Kawaji, Timo Lassmann, Matthias Harbers, Alistair RR Forrest, Vladimir B Bajic

**Affiliations:** 1 Computational Bioscience Research Center, Computer, Electrical and Mathematical Sciences and Engineering Division, King Abdullah University of Science and Technology (KAUST), Thuwal 23955-6900, Saudi Arabia; 2 Laboratory of Bioinformatics and Systems Biology, Engelhardt Institute of Molecular Biology, Russian Academy of Sciences, Vavilov str. 32, Moscow 119991GSP-1, Russia; 3 Department of Computational Systems Biology, Vavilov Institute of General Genetics, Russian Academy of Sciences, Gubkina str. 3, Moscow 119991, Russia; 4 RIKEN Omics Science Center, Yokohama, Kanagawa 230-0045, Japan; 5 RIKEN Center for Life Science Technologies, Division of Genomic Technologies, Yokohama, Kanagawa 230-0045, Japan; 6 RIKEN Preventive Medicine and Diagnosis Innovation Program, Wako, Saitama 351-0198, Japan

**Keywords:** DNA methylation, Transcription factor binding sites, Transcriptional regulation, CAGE, RRBS, CpG “traffic lights”, Bioinformatics, Computational biology

## Abstract

**Background:**

DNA methylation in promoters is closely linked to downstream gene repression. However, whether DNA methylation is a cause or a consequence of gene repression remains an open question. If it is a cause, then DNA methylation may affect the affinity of transcription factors (TFs) for their binding sites (TFBSs). If it is a consequence, then gene repression caused by chromatin modification may be stabilized by DNA methylation. Until now, these two possibilities have been supported only by non-systematic evidence and they have not been tested on a wide range of TFs. An average promoter methylation is usually used in studies, whereas recent results suggested that methylation of individual cytosines can also be important.

**Results:**

We found that the methylation profiles of 16.6% of cytosines and the expression profiles of neighboring transcriptional start sites (TSSs) were significantly negatively correlated. We called the CpGs corresponding to such cytosines “traffic lights”. We observed a strong selection against CpG “traffic lights” within TFBSs. The negative selection was stronger for transcriptional repressors as compared with transcriptional activators or multifunctional TFs as well as for core TFBS positions as compared with flanking TFBS positions.

**Conclusions:**

Our results indicate that direct and selective methylation of certain TFBS that prevents TF binding is restricted to special cases and cannot be considered as a general regulatory mechanism of transcription.

## Background

DNA methylation is one of the most studied epigenetic modifications. In differentiated cells in higher animals, methylated cytosine is almost always followed by guanine, associating methylation of 60-90% of all cytosines in a CpG context [1,2]. Although recent evidence showed that cytosine methylation in embryonic stem cells may also occur as CpHpG and CpHpH (where H corresponds to A, C, or T) [3-5], genome-wide distributions of cytosine methylation in CpHpG and especially in CpHpH have great variability between individuals, contrary to methylation in the CpG context, which demonstrates stable cell-type-specific methylation [4]. Thus, cell-type-specific regulatory patterns most likely depend on methylation in the CpG context.

Various methodologies have been developed to study DNA methylation at different genomic scales (for a review, see, for example, [6-8]) with direct sequencing of bisulfite-converted DNA [9] continuing to be the method of choice. However, the analysis of a single CpG site or a few CpG sites as surrogate indicators of DNA methylation status of the surrounding region is the most prevalent strategy in epigenetic studies at different scales, due to the assumption of the relatively homogeneous distribution of DNA methylation within genomic regions. This assumption is supported by multiple pieces of evidence of unmethylated CpGs closely co-located within CpG islands (CGIs) and methylated CpGs in repetitive elements. In addition, the level of methylation of the *HpaII* sites (CCGG) within CGIs demonstrates a correlation with average CGI methylation levels [10]. At the same time, methylated CpGs have been found in unmethylated CGIs [4]. It was also shown that a single differentially methylated CpG might affect transcription of the ESR1 gene [11]. Moreover, it was hypothesized that DNA methylation of CpG-rich and CpG-poor regions might be involved in different regulatory programs [12]. In short, whether or not the distinct methylation status of a single CpG affects specific transcription-related functions remains an open question.

It is widely accepted that cytosine methylation is a crucial regulatory mechanism in both normal and pathological processes. DNA methylation is involved in development [13,14], cellular differentiation [15], maintaining cellular identity [16], pluripotency [17], aging [18,19], memory formation [20], responses to environmental changes [21,22] and reactions to diet [23]. Several pathological conditions, including cancer [22,24], diabetes [25], Alzheimer’s and Parkinson’s diseases [26], also show aberrant DNA methylation. Profiles of DNA methylation can be inherited through cell division [16] and in some cases through generations [21]. However, recent studies of dynamic DNA methylation/de-methylation *in vivo*[27,28] challenge the conventional view that DNA methylation is a permanent epigenetic mark and suggest the possibility of exploring DNA methylation as a promising target for non-invasive therapies for diseases linked with aberrant methylation.

DNA methylation of gene promoters is tightly associated to the repression of transcription, yet the mechanisms are still unclear [29]. In the last four decades, multiple studies have shown that the level of DNA methylation in promoters is negatively correlated with the expression of downstream genes [30-35]. It was also hypothesized that ubiquitous, low-density cytosine methylation in vertebrate genomes can contribute to reduction of the transcriptional "noise" from inappropriate promoters [36]. Recently, multiple pieces of evidence arguing against the paradigm that DNA methylation always represses transcription have started to appear. Transcription of some genes was found to be independent of methylation [37]. Promoters with low CpG content are usually methylated, yet they still may be transcriptionally active [38,39]. Although intergenic and gene terminal CGIs are frequently methylated, they demonstrate a pervasive transcription [40]. Sparse DNA methylation of promoters may repress transcription, but this effect could be overcome by an enhancer [41]. Genes exhibiting high levels of promoter methylation during normal development remain suppressed in Dnmt1-deficient mouse embryos, suggesting that developmental gene control does not globally rely on cytosine methylation and that the effects of DNA methylation are limited to specialized processes such as imprinting and mobile elements repression [29]. Alternative promoter usage in different regions of the aged brain seems to be independent of promoter methylation [42]. Promoter sequences are able to recapitulate correct DNA methylation autonomously and demonstrate proper *de novo* methylation during differentiation in pluripotent cells independently of the transcriptional activity of corresponding downstream promoters [43]. Furthermore, in some cases, methylation is required for activation of transcription and therefore is positively correlated with gene expression [44].

**Figure 1:**
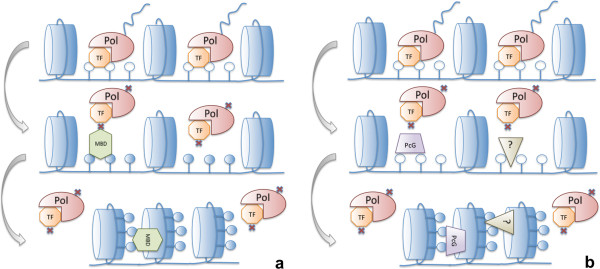
**Schematic representation of the interaction between promoter methylation and transcription of the gene.** In the absence of DNA methylation, TFs can bind DNA allowing RNA polymerase to bind and to start the transcription. Panel **a** shows the following scenario: if DNA becomes methylated, TFs are blocked from binding to DNA and therefore RNA polymerase is unable to bind and to initiate transcription. Panel **b** shows the following scenario: chromatin modifications reduce the ability of TFs to bind DNA and therefore RNA polymerase is unable to bind; the repressed condition of the chromatin is maintained by subsequent DNA methylation. PolII is shown as a maroon pie; nucleosome is shown as a blue cylinder. Plain (solid) lollipops represent unmethylated (methylated) cytosines. TF is shown as an orange octagon. The green hexagon and purple trapezoid are a methyl-binding domain and Policomb-group proteins, respectively. The brown triangle represents an unknown repressor.

Despite the various controversies, evidence that DNA methylation as an important step in regulation remains solid. The mechanisms of the interplay between methylation and expression are therefore critically important. It remains unclear whether DNA methylation is the cause or the consequence of altered gene expression. If DNA methylation causes gene repression, then there are several possible outcomes (Figure 1a). Cytosine methylation may directly affect the affinity of transcription factors (TFs) towards their binding sites (TFBSs) [45]. Non-systematic experimental evidence that DNA methylation can prevent binding of some TFs to particular TFBSs [45,46] supports this hypothesis. For example, methylation of the E-box (CACGTG) prevents n-Myc from binding to promoters of EGFR and CASP8 in a cell-specific manner [47]; methylation of the YY1-binding site in the promoter of the Peg3 gene represses the binding activity of YY1 *in vitro*[48]. It is also worth noting that experimentally determined TFBSs usually show low levels of DNA methylation [4,49,50] and that TF-TFBS recognition is often associated with the lack of methylation [51,52]. Furthermore, certain positions within CTCF binding sites are more sensitive to methylation than are others [53]. Methylated cytosine can also attract TFs, both activators [44,54] and repressors [55]. Methylation of the CRE sequence enhances the DNA binding of C/EBPα, which in turn activates a set of promoters specific for adipocyte differentiation [44,54]. Methyl-binding domain (MBD) proteins bind methylated CpG dinucleotide and induce histone deacetylation, subsequent chromatin condensation and gene repression [55].

The opposite scenario implies that chromatin modifications [56-58] reduce the accessibility of TFs and the transcriptional machinery to gene promoters, therefore leading to gene repression. DNA methylation in this model is not a cause, but a consequence of repression and serves to fix the repressed state of the chromatin (Figure 1b). In this case, cytosine methylation accumulates passively as a consequence of the independent absence of TF binding [50,53] or it appears as a result of direct DNA methyltransferase recruitment by transcription repression proteins such as the Polycomb group (PcG) protein EZH2 [59]. This model is supported by negative correlation of TF expression and average methylation of their TFBSs [50]. Besides, it was reported that binding of some TFs, including Sp1 and CTCF, is sufficient for maintaining a local unmethylated state [60-65]. Nevertheless, this scenario (Figure 1b) does not explain the sensitivity of certain TFs to methylation of their TFBSs.

In this study, we explore the evidence that supports one of these two scenarios. To achieve this, we first test whether methylation of a particular cytosine correlates with transcription. This effect may provide a basis for regulation of transcription through methylation of specific TFBSs. Second, we investigate whether some TFs are more sensitive than others to the presence of such cytosines in their TFBSs and what features of TFBSs can be associated with this sensitivity. To this end, we employed ENCODE [66] data on DNA methylation obtained by reduced representation bisulfite sequencing (RRBS) [67]. RRBS allows us to identify both methylated and unmethylated cytosines quantitatively at a single base pair resolution in the CCGG context in regions with high densities of rarely methylated cytosines, usually co-located within gene promoters [68]. To evaluate genome-wide expression across different cell types, we used FANTOM5 [69] data obtained by cap analysis of gene expression (CAGE) [70]. FANTOM5 provides quantitative estimation of expression in several hundreds of different cell types.

Our study shows that a fraction of single CpGs within promoters exhibits a significant negative correlation of their methylation profiles with the expression profiles of neighboring transcriptional start sites (TSSs) considered across various samples. Moreover, we observe a strong negative selection against the presence of such cytosines within TFBSs, especially in their core positions. Interestingly, we find that repressors are more sensitive to the presence of such cytosines in their binding sites.

This work is part of the FANTOM5 project. Data downloads, genomic tools and co-published manuscripts are collected at http://fantom.gsc.riken.jp/5/.

## Results and discussion

### Only a fraction of cytosines exhibits significant correlation between methylation and expression profiles of a corresponding TSS

It is well known that the level of cytosine methylation of promoters is negatively correlated with gene expression [71]; the role of methylation of particular CpGs in the regulation of gene expression has been demonstrated in the case of ESR1 [11]. The crucial role of the location of methylated regions relative to TSSs is also widely accepted. The question whether methylation of a particular cytosine may affect expression remains unanswered.

As the first step of this study, we studied whether the methylation level of a particular cytosine within a promoter region is correlated with the expression of the corresponding TSS, since such cytosines may serve as a basis for the regulation of transcription through TF binding. Table 1 demonstrates that among 237,244 cytosines analyzed in the study, only 16.6% (0.8%) have significantly (*P*-value ≤ 0.01) negative or positive Spearman Correlation Coefficients (*SCC*_*M/E*_) between methylation and expression profiles of a closely located TSS (see Methods). This sheds different light on the common perception of a link between methylation and gene expression. We call cytosines demonstrating significantly negative *SCC*_*M/E*_ CpG “traffic lights” (see Methods). In this study, we mostly focus on such cytosines.

**Table 1:** **Total numbers of CpGs with different SCC**_**M/E **_**between methylation and expression profiles**

**SCC**_**M/E **_**sign**	**SCC**_**M/E**_**,**	**SCC**_**M/E**_**,**	**SCC**_**M/E**_**,**	**SCC**_**M/E**_**,**	**SCC**_**M/E**_**,**	**SCC**_**M/E**_**,**
	***P*-value ≤ 0.05**	***P*-value ≤ 0.01**	***P*-value ≤ 0.001**	***P*-value ≤ 0.05, fraction**	***P*-value ≤ 0.01, fraction**	***P*-value ≤ 0.001, fraction**
Negative	73328	39414	17031	0.309	0.166	0.072
Positive	5750	1832	479	0.024	0.008	0.002

The total number of CpGs in the study is 237,244.

Out of 50 cell types analyzed in this study, 14 were malignant. Genome-wide DNA methylation in cancer cells is dramatically different from that in normal cells (for the review see, for example [72-75]). Although we believe that the basic mechanism of interaction between DNA methylation and expression should be the same in cancer and non-cancer cells, we repeated the experiments on the 36 normal cell types and obtained similar results (Additional file 1): only a small fraction (9.5% and 1.5%) of cytosines have significant (*P*-value ≤ 0.01) negative and positive *SCC*_*M/E*_, respectively.

CAGE tags are often found within gene bodies [76] and methylation of a gene body may have a positive correlation with gene expression [77-79]. It was also suggested that the cytosines within gene bodies are often not methylated (5mC) but hydroxymethylated (5hmC) [80]. However, bisulfite-based methods of cytosine modification detection (including RRBS) are unable to distinguish these two types of modifications [81]. The presence of 5hmC in a gene body may be the reason why a fraction of CpG dinucleotides has a significant positive *SCC*_*M/E*_ value. Unfortunately, data on genome-wide distribution of 5hmC in humans is available for a very limited set of cell types, mostly developmental [82,83], preventing us from a direct study of the effects of 5hmC on transcription and TFBSs. At the current stage the 5hmC data is not available for inclusion in the manuscript. Yet, we were able to perform an indirect study based on the localization of the studied cytosines in various genomic regions. We tested whether cytosines demonstrating various *SCC*_*M/E*_ are co-located within different gene regions (Table 2). Indeed, CpG “traffic lights” are located within promoters of GENCODE [84] annotated genes in 79% of the cases, and within gene bodies in 51% of the cases, while cytosines with positive *SCC*_*M/E*_ are located within promoters in 56% of the cases and within gene bodies in 61% of the cases. Interestingly, 80% of CpG “traffic lights” are located within CGIs, while this fraction is smaller (67%) for cytosines with positive *SCC*_*M/E*_. This observation allows us to speculate that CpG “traffic lights” are more likely methylated, while cytosines demonstrating positive *SCC*_*M/E*_ may be subject to both methylation and hydroxymethylation. Cytosines with positive and negative *SCC*_*M/E*_ may therefore contribute to different mechanisms of epigenetic regulation. It is also worth noting that cytosines with insignificant (*P*-value > 0.01) *SCC*_*M/E*_ are more often located within the repetitive elements and less often within the conserved regions and that they are more often polymorphic as compared with cytosines with a significant *SCC*_*M/E*_, suggesting that there is natural selection protecting CpGs with a significant *SCC*_*M/E*_.

**Table 2:** **Fraction of cytosines demonstrating different SCC**_**M/E **_**within genome regions**

	**CGI**	**Gene promoters**	**Gene bodies**	**Repetitive elements**	**Conserved regions**	**SNP**	**DNase sensitivity regions**
CpG “traffic lights”	0.801	0.793	0.507	0.095	0.203	0.008	0.926
SCC_M/E_ > 0	0.674	0.556	0.606	0.095	0.210	0.009	0.829
SCC_M/E_ insignificant	0.794	0.733	0.477	0.128	0.198	0.010	0.897

### Selection against TF binding sites overlapping with CpG “traffic lights”

We hypothesize that if CpG “traffic lights” are not induced by the average methylation of a silent promoter, they may affect TF binding sites (TFBSs) and therefore may regulate transcription. It was shown previously that cytosine methylation might change the spatial structure of DNA and thus might affect transcriptional regulation by changes in the affinity of TFs binding to DNA [47-49]. However, the answer to the question of if such a mechanism is widespread in the regulation of transcription remains unclear. For TFBSs prediction we used the remote dependency model (RDM) [85], a generalized version of a position weight matrix (PWM), which eliminates an assumption on the positional independence of nucleotides and takes into account possible correlations of nucleotides at remote positions within TFBSs. RDM was shown to decrease false positive rates effectively as compared with the widely used PWM model.

**Figure 2:**
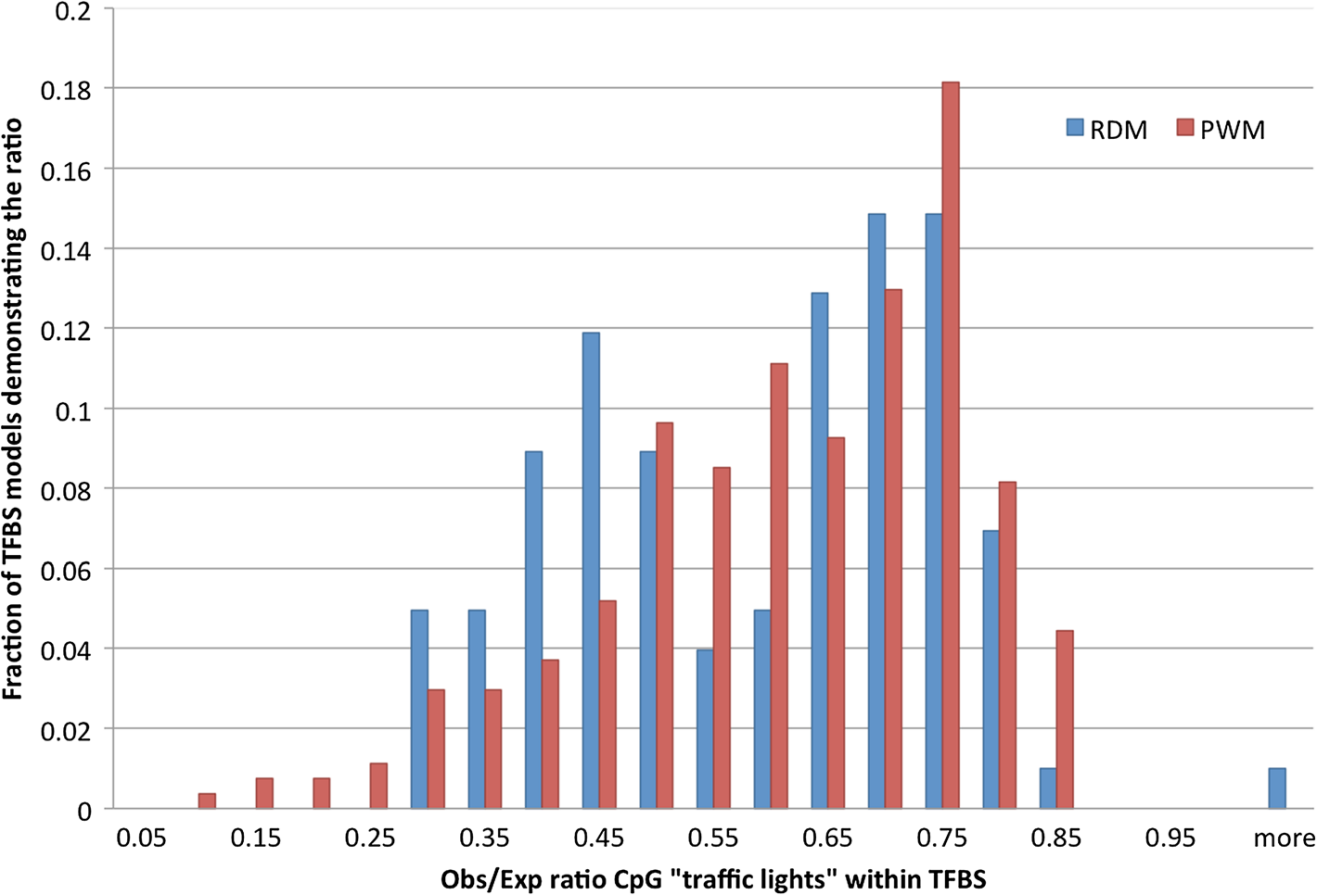
**Distribution of the observed number of CpG “traffic lights” to their expected number overlapping with TFBSs of various TFs.** The expected number was calculated based on the overall fraction of significant (*P*-value < 0.01) CpG “traffic lights” among all cytosines analyzed in the experiment.

Our results demonstrate (Additional file 2) that from the 271 TFs studied here (having at least one CpG “traffic light” within TFBSs predicted by RDM), 100 TFs had a significant underrepresentation of CpG “traffic lights” within their predicted TFBSs (*P*-value < 0.05, Chi-square test, Bonferoni correction) and only one TF (OTX2) had a significant overrepresentation of CpG “traffic lights” within the predicted TFBSs. Similar results were obtained using only the 36 normal cell lines: 35 TFs had a significant underrepresentation of CpG “traffic lights” within their predicted TFBSs (*P*-value < 0.05, Chi-square test, Bonferoni correction) and no TFs had a significant overrepresentation of such positions within TFBSs (Additional file 3). Figure 2 shows the distribution of the observed-to-expected ratio of TFBS overlapping with CpG “traffic lights”. It is worth noting that the distribution is clearly bimodal with one mode around 0.45 (corresponding to TFs with more than double underrepresentation of CpG "traffic lights" in their binding sites) and another mode around 0.7 (corresponding to TFs with only 30% underrepresentation of CpG “traffic lights” in their binding sites). We speculate that for the first group of TFBSs, overlapping with CpG “traffic lights” is much more disruptive than for the second one, although the mechanism behind this division is not clear.

To ensure that the results were not caused by a novel method of TFBS prediction (i.e., due to the use of RDM), we performed the same analysis using the standard PWM approach. The results presented in Figure 2 and in Additional file 4 show that although the PWM-based method generated many more TFBS predictions as compared to RDM, the CpG “traffic lights” were significantly underrepresented in the TFBSs in 270 out of 279 TFs studied here (having at least one CpG “traffic light” within TFBSs as predicted by PWM), supporting our major finding.

We also analyzed if cytosines with significant positive *SCC*_*M/E*_ demonstrated similar underrepresentation within TFBS. Indeed, among the tested TFs, almost all were depleted of such cytosines (Additional file 2), but only 17 of them were significantly over-represented due to the overall low number of cytosines with significant positive *SCC*_*M/E*_. Results obtained using only the 36 normal cell lines were similar: 11 TFs were significantly depleted of such cytosines (Additional file 3), while most of the others were also depleted, yet insignificantly due to the low number of total predictions. Analysis based on PWM models (Additional file 4) showed significant underrepresentation of such cytosines for 229 TFs and overrepresentation for 7 (DLX3, GATA6, NR1I2, OTX2, SOX2, SOX5, SOX17). Interestingly, these 7 TFs all have highly AT-rich binding sites with very low probability of CpG.

It was previously shown that cytosine methylation can prevent binding of several TFs (such as Sp1 [60], CTCF [53] and others) and, therefore, methylation may serve as a global regulatory mechanism for cell-specific TF binding. Yet, we observe that most of TFs avoid CpG “traffic lights” in their binding sites, suggesting a potentially damaging effect of CpG “traffic lights” to TFBS and therefore a natural selection against TFBS overlapping with CpG “traffic lights”.

Computational prediction of TFBSs identifies DNA regions of potential binding, which may not be available for a TF in a particular cell type due to chromatin modifications. To avoid a bias caused by potential TFBSs that are not functional in particular cell types, we used experimentally obtained regions of TF binding. Chromatin immunoprecipitation followed by parallel DNA sequencing (ChIP-seq) is an effective experimental technique for the identification of regions for DNA-protein interaction [86]. Yet, regions where TFs most likely bind DNA (ChIP-seq peaks) in a particular cell type are relatively long, usually longer than several hundreds of base pairs, while real TFBSs are on average a dozen base pairs long. Therefore, we combined experimental and computational approaches and filtered out the predictions of TFBSs outside of ChiP-seq peak regions. We tested our results on ChIP-seq data for CTCF as it is the only TF in ENCODE with experimental binding information in as many as 22 cell types out of the 50 cell types we used in our study (14 of the 22 were normal cell types). Results in Additional file 5 support our initial finding: CTCF binding sites avoid CpG “traffic lights”. ChIP-seq data for other TFs are available only for the cancer cell lines included in our study, making it impossible to draw conclusions about normal cell functioning. At the current stage the ChiP-seq data for other TFs is not available for inclusion in the manuscript. Our findings suggest that changing a TF’s affinity to DNA or even blocking TF binding sites by direct and selective methylation is limited to certain TFBSs within a few promoters and thus is not likely to be a general mechanism of methylation-dependent regulation of gene expression.

### TFBSs of repressors are especially sensitive to the presence of CpG “traffic lights”

Overlapping of TFBS with CpG “traffic lights” may affect TF binding in various ways depending on the functions of TFs in the regulation of transcription. There are four possible simple scenarios, as described in Table 3. However, it is worth noting that many TFs can work both as activators and repressors depending on their cofactors. Moreover, some TFs can bind both methylated and unmethylated DNA [87]. Such TFs are expected to be less sensitive to the presence of CpG “traffic lights” than are those with a single function and clear preferences for methylated or unmethylated DNA.

**Table 3:** **Expected sign of *SCC***_***M/E ***_**depending on TF binding preferences and function**

**TF binding preferences**			
TF function	Unmethylated DNA	Methylated DNA	Both
Activator	(1) negative *SCC*_*M/E*_	(2) positive *SCC*_*M/E*_	insignificant *SCC*_*M/E*_
Repressor	(3) positive *SCC*_*M/E*_	(4) negative *SCC*_*M/E*_	insignificant *SCC*_*M/E*_
Both	insignificant *SCC*_*M/E*_	insignificant *SCC*_*M/E*_	

There are four possible scenarios of interaction of DNA methylation and TF functions:

(1) TF can bind unmethylated DNA and cannot bind methylated DNA. TF acts as a transcription activator. The methylation profile of cytosines within TFBS should be negatively correlated with TSS expression.

(2) TF can bind methylated DNA and cannot bind unmethylated DNA. TF acts as a transcription activator. The methylation profile of cytosines within TFBS should be positively correlated with TSS expression.

(3) TF can bind unmethylated DNA and cannot bind methylated DNA. TF acts as a transcription repressor. The methylation profile of cytosines within TFBS should be positively correlated with TSS expression.

(4) TF can bind methylated DNA and cannot bind unmethylated DNA. TF acts as transcription repressor. The methylation profile of cytosines within TFBS should be negatively correlated with TSS expression.

**Figure 3:**
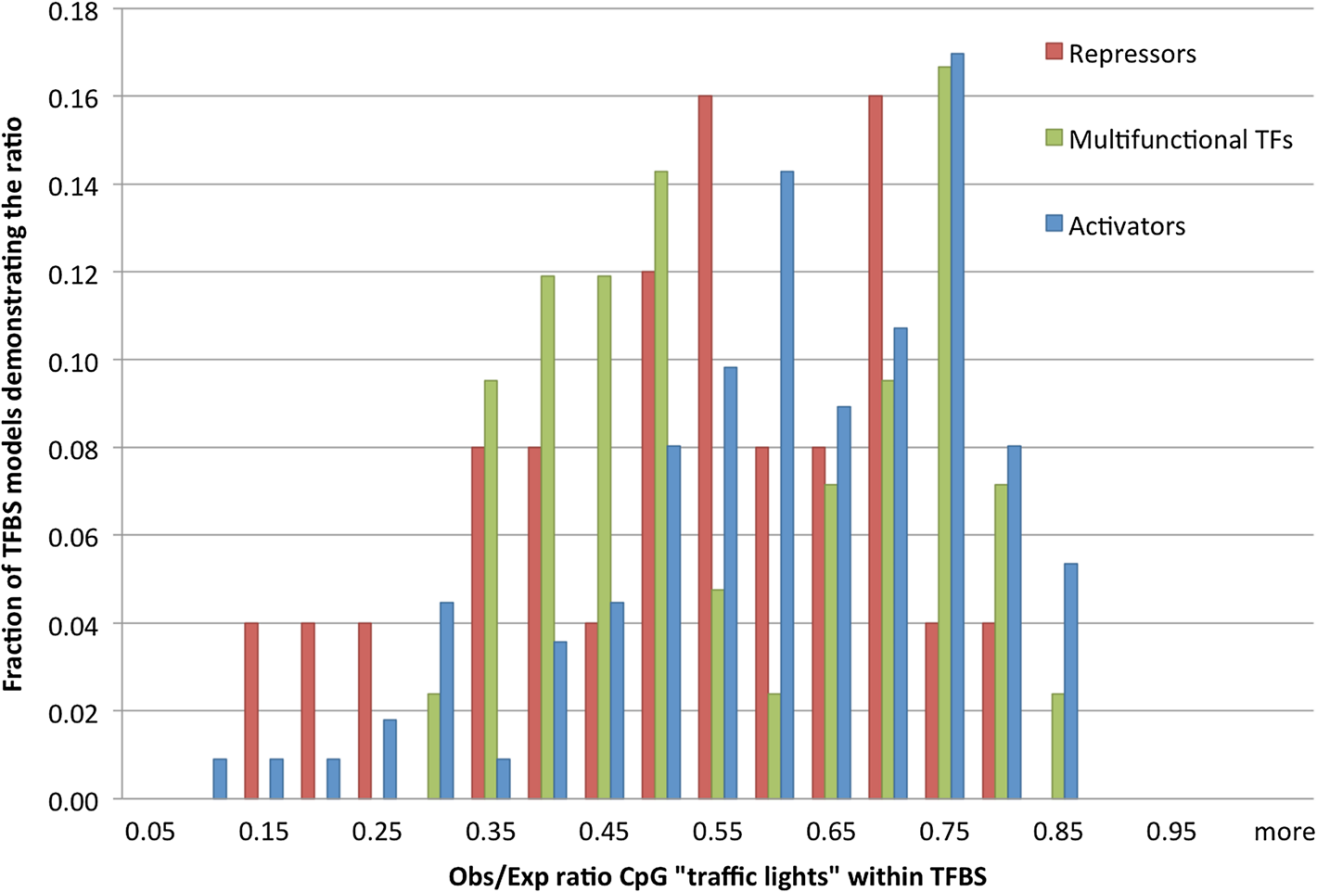
**Distribution of the observed number of CpG “traffic lights” to their expected number overlapping with TFBSs of activators, repressors and multifunctional TFs.** The expected number was calculated based on the overall fraction of significant (*P*-value < 0.01) CpG “traffic lights” among all cytosines analyzed in the experiment.

Using information about molecular function of TFs from UniProt [88] (Additional files 2, 3, 4 and 5), we compared the observed-to-expected ratio of TFBS overlapping with CpG “traffic lights” for different classes of TFs. Figure 3 shows the distribution of the ratios for activators, repressors and multifunctional TFs (able to function as both activators and repressors). The figure shows that repressors are more sensitive (average observed-to-expected ratio is 0.5) to the presence of CpG “traffic lights” as compared with the other two classes of TFs (average observed-to-expected ratio for activators and multifunctional TFs is 0.6; t-test, *P*-value < 0.05), suggesting a higher disruptive effect of CpG “traffic lights” on the TFBSs of repressors. Although results based on the RDM method of TFBS prediction show similar distributions (Additional file 6), the differences between them are not significant due to a much lower number of TFBSs predicted by this method. Multifunctional TFs exhibit a bimodal distribution with one mode similar to repressors (observed-to-expected ratio 0.5) and another mode similar to activators (observed-to-expected ratio 0.75). This suggests that some multifunctional TFs act more often as activators while others act more often as repressors. Taking into account that most of the known TFs prefer to bind unmethylated DNA, our results are in concordance with the theoretical scenarios presented in Table 3.

### “Core” positions within TFBSs are especially sensitive to the presence of CpG “traffic lights”

We also evaluated if the information content of the positions within TFBS (measured for PWMs) affected the probability to find CpG “traffic lights” (Additional files 7 and 8). We observed that high information content in these positions (“core” TFBS positions, see Methods) decreases the probability to find CpG “traffic lights” in these positions supporting the hypothesis of the damaging effect of CpG “traffic lights” to TFBS (t-test, *P*-value < 0.05). The tendency holds independent of the chosen method of TFBS prediction (RDM or RWM). It is noteworthy that “core” positions of TFBS are also depleted of CpGs having positive *SCC*_*M/E*_ as compared to “flanking” positions (low information content of a position within PWM, (see Methods), although the results are not significant due to the low number of such CpGs (Additional files 7 and 8).

## Conclusions

We found that the methylation profiles and expression profiles in 16.6% of single CpG dinucleotides in CAGE-derived promoters were significantly negatively correlated with neighbouring TSS, supporting the argument that single cytosine methylation is involved in the regulation of transcription. In a way, the current common perception of the link between methylation and gene expression is seen in a different light. Unexpectedly, we observed a strong selection against the presence of CpG “traffic lights” within the TFBSs of many TFs. We demonstrated that the selection against CpG “traffic lights” within TFBS is even more pronounced in the case of “core” positions within TFBSs as compared to “flanking” positions. These observations allow us to suggest that blocking of TFBSs by selective methylation is unlikely to be a general mechanism of methylation-dependent transcription regulation and that such a mechanism is limited to special cases. We conclude that the regulation of expression via DNA methylation and via TF binding are relatively independent regulatory mechanisms; both mechanisms are thus not in a direct causal relationship. Known cases of interaction between these mechanisms appear mostly because they operate on the same target regions (promoters) and require intermediate partners, for example, modification of chromatin.

## Methods

### Cell types

We manually selected 137 FANTOM5 samples (cell types) matching 50 ENCODE samples. We grouped them into 50 classes of identical or similar biological cell types. To reduce the noise coming from inexact matching of cell types between FANTOM5 and ENCODE data, we averaged the expression/methylation values for different technical or biological replicas, donors and cell types within the same class. Detailed information is provided in Additional file 9.

All human samples used in the FANTOM5 project were either exempted material (available in public collections or commercially available), or provided under informed consent. All non-exempt material is covered under RIKEN Yokohama Ethics applications (H17-34 and H21-14) and collected in compliance with the Helsinki Declaration.

### TSSs and promoter regions

We used TSSs found by the CAGE method in FANTOM5. The relative log expression normalization method (RLE [89]) was applied to CAGE-tags in each sample [69]. For a particular TSS, we referred to a set of expression values across the selected 50 classes of cell types as an expression profile. Low expressed CAGE-tag clusters may be non-robust to sequencing errors or heterogeneity of the cell population. To reduce the effect of such CAGE-tag clusters, we excluded TSSs with all RLE-normalized expression values less than 1. For each CAGE-tag cluster, we selected a promoter region of 1500 bp upstream and 500 bp downstream of the ends of reported CAGE-tag clusters. Overlapping promoters were considered independently.

### Cytosine methylation data

We used cytosine methylation data obtained by RRBS (http://genome.ucsc.edu/cgi-bin/hgFileUi?db=hg19&g=wgEncodeHaibMethylRrbs). All data included cytosine methylation only in the CCGG context. We excluded cytosines covered by less than 10 reads. For a particular cytosine, we referred to a set of methylation values (the proportion of methylated reads relative to all reads) across the selected 50 cell types as a methylation profile. We excluded cytosines having methylation data for less than 50% of samples (25 when using all 50 cell types and 18 when using the 36 normal cell types) in the methylation profiles.

While each particular cytosine may be either methylated or unmethylated, the RRBS technique measures the average methylation of a particular cytosine in the cell population, which results in a 0 to 100% range of values. Although methylation values of most of the cytosines tend to be 0 or 100%, intermediate values are also possible. Low (but not 0) levels of cytosine methylation may appear as a result of experimental errors, and these levels can affect further analysis. To avoid any bias caused by such cytosines, we used only positions differentially methylated between cell types. We defined a CpG as differentially methylated if the amplitude (the difference between the maximum and minimum values in the normalized profile) of the methylation profile for a particular CpG was greater than 50%.

### Correlation of cytosine methylation and TSS expression

For all cytosines located within promoter regions, we calculated the Spearman Correlation Coefficient between methylation profiles of the cytosine and the expression profiles of the corresponding TSS (referred to as *SCC*_*M/E*_). We estimated the statistical significance of *SCC*_*M/E*_ based on transformation to a Student’s t-test distribution:

$$t\;=\;\mathrm{SC}C_{M/E}\sqrt{\frac{n-2}{1-\mathrm{SC}C_{M/E}^{2}}}$$

Here *n* is the length of the methylation/expression profile for a given position. In our analysis (if not stated otherwise), we referred to positions with *P*-values (SCC_M/E_) ≤ 0.01 as positions with significantly negative or positive correlations between the methylation and the expression profiles. It is noteworthy that due to the overlapping of promoter regions for different TSSs, one cytosine may have several *SCC*_*M/E*_. In the case of overlapping promoters, it is difficult to estimate which TSS is affected by the methylation of a particular cytosine. We therefore considered that a particular CpG affects transcription if it has at least one *SCC*_*M/E*_ above (or below) the significance level (see Table 1).

### CpG “traffic lights”

To avoid bias in estimating *SCC*_*M/E*_ for low methylated cytosines caused by experimental errors, we introduced differentially methylated cytosines based on the difference between the highest and lowest value (amplitude) in the normalized methylated profile when it was greater than 50% of the maximum possible value. In the analysis of TFBSs affected by cytosine methylation, we considered only CpGs differentially methylated across cell types. We introduced the term CpG “traffic lights” to describe differentially methylated cytosines with significantly (*P*-values (SCC_M/E_) ≤ 0.01) negative *SCC*_*M/E*_.

We also looked for co-localization of CpG “traffic lights” and several genomic features (data downloaded from http://hgdownload.soe.ucsc.edu/goldenPath/hg19/database/): known gene promoters (1500 bp upstream of TSS and 500 bp downstream) and gene bodies (500 bp downstream TSS to the end of the gene) (wgEncodeGencodeBasicV140); CpG islands (cpgIslandExt); DNase sensitivity regions (wgEncodeRegDnaseClusteredV2); repetitive elements (rmsk); SNPs (snp137Common); and conserved elements (phastConsElements46wayPrimates).

### Prediction of TFBSs using the remote dependency models

To create RDMs, we used binding site alignments from HOCOMOCO [90]. This collection of TFBS models was selected due to the low level of redundancy of TFBS models per single TF. Binding sites having scores less than PWM thresholds were excluded. PWM thresholds were selected according to the *P*-value < 0.0005 (i.e., when 5 of 10,000 random words had scores no less than the thresholds). *P*-values were computed by the MACRO-APE software (http://autosome.ru/macroape) [90] that implements the strategy presented in the work of Touzet and Varre [91]. Due to the large number of parameters in RDM models as compared to PWM models provided in HOCOMOCO, the minimal number of sequences in the alignment was increased from 8 to 15. Filtered alignments of fewer than 15 binding sites were discarded, which reduced the initial set of 426 TFBS models available in HOCOMOCO to 280 TFBS models (Additional file 4, column 1).

Using the frequency of each dinucleotide with one nucleotide being at position *i* and the other at position *j*, where *i* = 1, …, *L*-1, *j* = *i* + 1, …, *L,* in the set of aligned binding sites, the dinucleotide frequency matrix with remote dependencies was constructed and normalized similar to PWM normalization in Bajic *et al.*[92]:

$$\mathrm{RD}M_{a,i,j}=\frac{f_{a,i,j}}{\sum_{i=1}^{L-1}\sum_{j=i+1}^{L}\max_{a}\left(f_{a,i,j}\right)}$$

Here *f*_*a,i,j*_ is the frequency of dinucleotide *a* formed of nucleotides at positions *i* and *j*, and *L* is the length of the aligned TFBSs. We predicted TFBSs using the RDM models across the whole promoter set.

### Prediction of TFBSs using position weight matrices

To check if the TFBS prediction method affects the results, we also predicted TFBS using widely accepted PWM models. We took the same PWMs from HOCOMOCO as used for RDM construction. PWM thresholds were selected according to the *P*-value of 0.0005 (Additional file 10).

### TFBSs potentially affected by DNA methylation

We selected all cytosines for which *SCC*_*M/E*_ were available and checked whether they were located within predicted TFBSs. The total number of predicted TFBSs is available in Additional files 2, 3 and 4 (column D). It is noteworthy that average GC-content of the RDM hits was undistinguishable from that of the binding sites in the initial alignments.

### “Core” and “flanking” CpG positions within TFBS

If we consider all genome-wide hits of any TFBS model, we may find that CpG dinucleotides can appear almost in every position of TFBSs. However, some positions within binding sites contain CpG dinucleotide more often than do others, so we repeated the analysis for each type of binding site position separately. For a particular TFBS model, we selected CpG positions in the HOCOMOCO alignments according to the information content of the corresponding PWM columns. Information content is defined as DIC (Discrete Information Content [93]) separately for different types of binding site positions. For a particular TFBS model, we selected CpG positions in the HOCOMOCO alignments according to the information content of the corresponding PWM columns:

$$\mathrm{DI}C_{j}=\frac{1}{N}\left(\sum_{a\in \left(A,C,G,T\right)}\log\left(x_{a,j}!\right)-\log\left(N!\right)\right),$$

Here *x*_*a,j*_ are elements of the position count matrix (i.e., nucleotide counts), *N* is the total number of aligned TFBS sequences. In contrast to classic information content [94], DIC is based on raw counts (instead of per-column nucleotide probabilities, which can be inaccurate for a small set of aligned sequences). We define two empirical DIC thresholds [95]*Th* and *th* (introduced in [96]). *Th* corresponds to the DIC of the column having only 3 (of 4 possible) nucleotides that have the same frequency, *th* corresponds to the DIC of the column having two nucleotides with the same frequency, *f*, and the other two nucleotides each with the frequency *2f*.

The CpG positions have C and G as major nucleotides (with the highest frequency) in the neighbouring columns. High information content CpG (“core” TFBS positions) has both C and G columns with DIC greater than *Th*. The medium (or low) information content CpG (“flanking” TFBS positions) has both C- and G-column DIC between *Th* and *th* (or lower than *th*). The summary is presented in Additional files 4 and 5.

## Abbreviations

RRBS: Reduced representation bisulphite sequencing; CAGE: Cap analysis of gene expression; ChIP-seq: Chromatin immunoprecipitation followed by DNA sequencing; TSS: Transcription start site; TF: Transcription factor; TFBS: Transcription factor binding site; RDM: Remote dependency model; PWM: Position weight matrix; SCCM/E: Spearman correlation coefficient between methylation and expression profiles; CGI: CpG island; DIC: Discrete information content.

## Competing interests

The authors declare that they have no competing interests.

## Authors’ contributions

YAM designed the computational experiments, selected and preprocessed the data, performed statistical analysis and wrote the manuscript. AK performed most of the data analysis. WBA and MdSIB provided RDM models and tools for threshold estimation and mapping. TL was responsible for tag mapping. HK managed the data handling. ARRF was responsible for FANTOM5 management and its concept. IVK performed part of the analysis and contributed to the design of the experiments and writing of the manuscript. VBB contributed to the design of the experiments and writing of the manuscript. All authors read and approved the final manuscript.

## Supplementary Material

Additional file 1 **Contains the total number of analyzed CpGs as well as the count of CpG demonstrating SCC**_**M/E **_**above certain significance levels. These results were obtained using only the 36 normal cell types.** Click here for file

Additional file 2 **Contains RDM-based predicted TFBSs based on 50 cell samples; tables containing information about the names of the TFBSs models used, their function in regulation (activator or repressor), the number of cytosines in our study (with any *SCC***_***M/E***_**) overlapping with TFBSs, the number of CpG “traffic lights” overlapping with TFBSs for each TF, the expected number of such overlaps and the statistical significance of the over-/underrepresentation of TFBS in CpG “traffic light” positions.** Consistent information is given for positions with positive *SCC*_*M/E*_*.* Click here for file

Additional file 3 **Contains RDM-based predicted TFBSs based on the 36 normal cell samples; tables containing information about the names of the TFBSs models used, their function in regulation (activator or repressor), the number of cytosines in our study (with any *SCC***_***M/E***_**) overlapping with TFBSs, the number of CpG “traffic lights” overlapping with TFBSs for each TF, the expected number of such overlaps and the statistical significance of the over-/underrepresentation of TFBS in CpG “traffic light” positions.** Consistent information is given for positions with positive *SCC*_*M/E*_*.* Click here for file

Additional file 4 **Contains PWM-based predicted TFBSs based on 50 cell samples; tables containing information about the names of the TFBSs models used, their function in regulation (activator or repressor), the number of cytosines in our study (with any *SCC***_***M/E***_**) overlapping with TFBSs, the number of CpG “traffic lights” overlapping with TFBSs for each TF, the expected number of such overlaps and the statistical significance of the over-/underrepresentation of TFBS in CpG “traffic light” positions.** Consistent information is given for positions with positive *SCC*_*M/E*_*.* Click here for file

Additional file 5 **Contains RDM-based predicted TFBS for CTCF supported with ChIP-seq peak data; tables containing information about the names of the TFBSs models used, their function in regulation (activator or repressor), the number of cytosines in our study (with any *SCC***_***M/E***_**) overlapping with TFBSs, the number of CpG “traffic lights” overlapping with TFBSs for each TF, the expected number of such overlaps and the statistical significance of the over-/underrepresentation of TFBS in CpG “traffic light” positions.** Consistent information is given for positions with positive *SCC*_*M/E*_*.* Click here for file

Additional file 6 **Contains a figure showing the distribution of the observed to expected ratio of CpG “traffic lights” overlapping with TFBSs of activators, repressors and multifunctional TFs.** TFBSs were predicted using RDM. Click here for file

Additional file 7 **Contains RDM-based predicted TFBSs. Tables containing the positions within TFBSs with high, medium and low IC, the number of cytosines in our study (with any *SCC***_***M/E***_**) overlapping with TFBS, and the number of CpG “traffic lights” overlapping with TFBS for each TF.** Consistent information is given for positions with positive *SCC*_*M/E*_*.* Click here for file

Additional file 8 **Contains PWM-based predicted TFBSs.** Tables containing the positions within TFBSs with high, medium and low IC, the number of cytosines in our study (with any *SCC*_*M/E*_) overlapping with TFBS, and the number of CpG “traffic lights” overlapping with TFBS for each TF. Consistent information is given for positions with positive *SCC*_*M/E*_*.* Click here for file

Additional file 9 **Contains a table listing FANTOM5 samples (cell types) matching 50 ENCODE samples.** We grouped them into 50 classes of identical or similar biological cell types. The ENCODE sample description is also provided. Normal/cancer cell types (36/14) are marked in the last column. Click here for file

Additional file 10 **Contains thresholds for PWM corresponding to the *P*-value < 0.0005 (i.e., when 5 of 10,000 random words have scores no less than the thresholds).***P*-values were computed by MACRO-APE software (http://autosome.ru/macroape). Click here for file
