# Supplementary material for: Effects of cytosine methylation on transcription factor binding sites
Source: BMC Genomics. 2014 Mar 26;15:119. doi: 10.1186/1471-2164-15-119 (PMC3986887; doi:10.1186/1471-2164-15-119)
Supplement: Additional file 1 — Contains the total number of analyzed CpGs as well as the count of CpG demonstrating SCC M/E above certain significance levels. These results were obtained using only the 36 normal cell types. [file 1471-2164-15-119-S1.docx]

Table S1: Total number of CpGs and SCCM/E between methylation and expression profiles

| SCCM/E  sign | Total  number  of  cytosines | SCCM/E,  p-value ≤  0.05 | SCCM/E,  p-value ≤  0.01 | SCCM/E,  p-value ≤  0.001 | SCCM/E,  p-value ≤  0.05,  fraction | SCCM/E,  p-value ≤  0.01,  fraction | SCCM/E,  p-value ≤  0.001,  fraction |
| --- | --- | --- | --- | --- | --- | --- | --- |
| Negative | 75335 | 14676 | 7173 | 3092 | 0.195 | 0.095 | 0.041 |
| Positive |  | 3396 | 1153 | 304 | 0.045 | 0.015 | 0.004 |
