# Supplementary figures and images for: Effects of cytosine methylation on transcription factor binding sites
Source: BMC Genomics. 2014 Mar 26;15:119. doi: 10.1186/1471-2164-15-119 (PMC3986887; doi:10.1186/1471-2164-15-119)

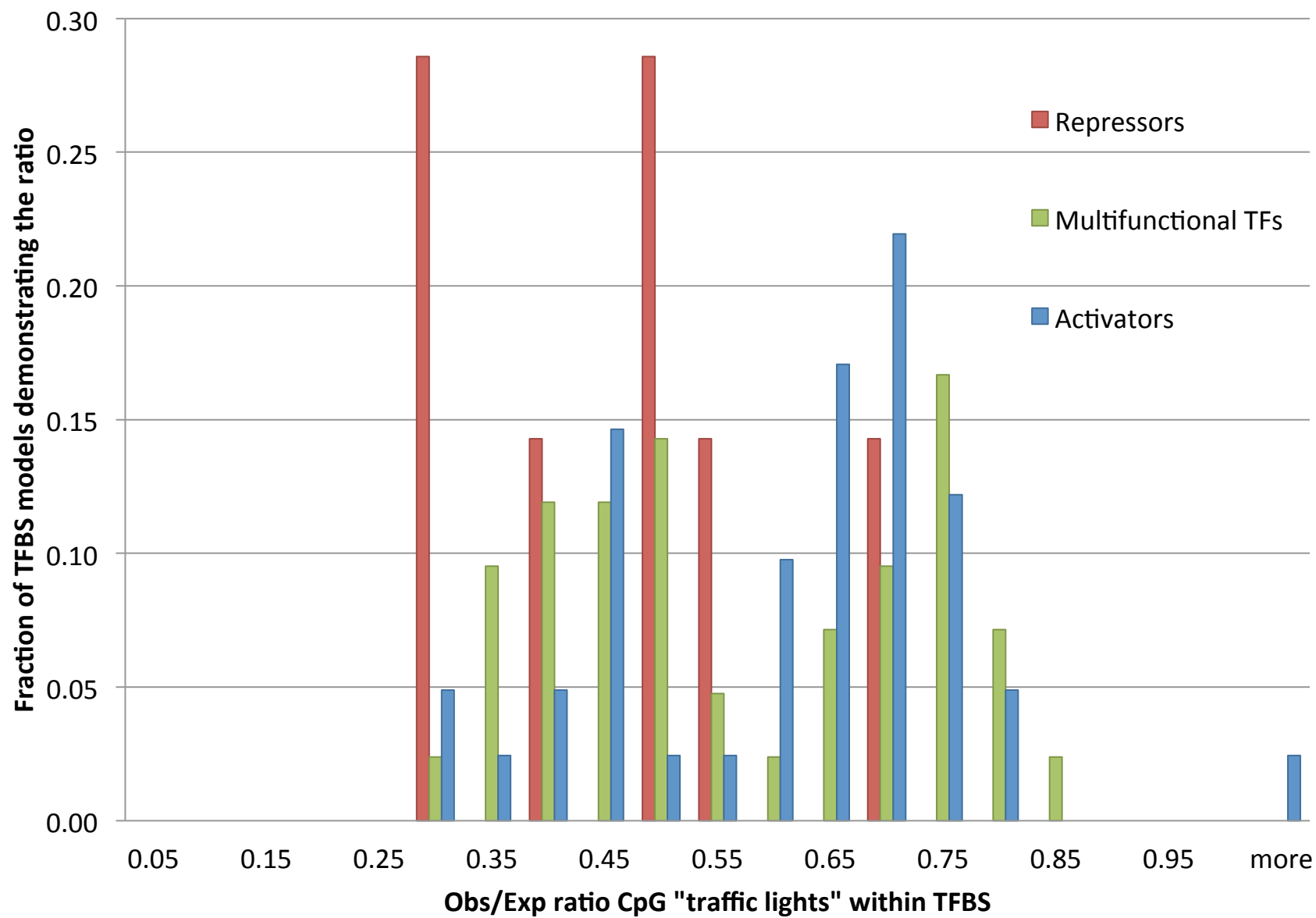

Supplement: Additional file 6 — Contains a figure showing the distribution of the observed to expected ratio of CpG “traffic lights” overlapping with TFBSs of activators, repressors and multifunctional TFs. TFBSs were predicted using RDM. [file 1471-2164-15-119-S6.pdf]
